# Simultaneous Target Classification and Moving Direction Estimation in Millimeter-Wave Radar System

**DOI:** 10.3390/s21155228

**Published:** 2021-08-02

**Authors:** Jin-Cheol Kim, Hwi-Gu Jeong, Seongwook Lee

**Affiliations:** School of Electronics and Information Engineering, College of Engineering, Korea Aerospace University, Goyang-si 10540, Gyeonggi-do, Korea; wls12cjf@kau.kr (J.-C.K.); wjdgnlrn02@kau.kr (H.-G.J.)

**Keywords:** millimeter-wave radar, moving direction estimation, target classification, you only look once (YOLO)

## Abstract

In this study, we propose a method to identify the type of target and simultaneously determine its moving direction in a millimeter-wave radar system. First, using a frequency-modulated continuous wave (FMCW) radar sensor with the center frequency of 62 GHz, radar sensor data for a pedestrian, a cyclist, and a car are obtained in the test field. Then, a You Only Look Once (YOLO)-based network is trained with the sensor data to perform simultaneous target classification and moving direction estimation. To generate input data suitable for the deep learning-based classifier, a method of converting the radar detection result into an image form is also proposed. With the proposed method, we can identify the type of each target and its direction of movement with an accuracy of over 95%. Moreover, the pre-trained classifier shows an identification accuracy of 85% even for newly acquired data that have not been used for training.

## 1. Introduction

One of the essential functions required for autonomous vehicles is to recognize and identify various objects on the road. In general, by processing data acquired from automotive sensors, such as cameras, lidars, and radars, the type and location information of an object can be estimated. For example, the vision sensor can detect lanes [1] and discriminate objects on the road [2]. In addition, the lidar and radar sensors mainly perform the function of estimating the position of an object [3,4]. However, in dark nights or in bad weather conditions, the object recognition performance of the camera is seriously deteriorated. Thus, there is a need for a method that can compensate for the degradation of the camera’s recognition performance using other automotive sensors.

In general, the radar’s detection performance is not severely degraded even in the harsh environments [5]. Moreover, the disadvantages of conventional automotive radar sensors having low range and angular resolution are overcome by using high bandwidths and multiple antenna elements [6]. Based on these advances, it has become possible to detect objects on the road in high resolution even through automotive radar sensors. In high-resolution radar systems, an object that was detected as a single point is detected as a point cloud composed of multiple points [7], which implies that not only the location information of the object but also its size and shape can be estimated. Therefore, it becomes possible to effectively classify the types of detected objects in the high-resolution radar system. The target classification is important in determining the control principles of autonomous vehicles.

In this study, we propose a method to discriminate the type of object in a high-resolution radar system. Even the moving direction of the detected object can be estimated simultaneously through the proposed method. In automotive radar systems, the possibility of identifying the type of detected object by applying deep learning techniques has been confirmed in many studies [8,9]. In addition, a method of estimating the moving direction of a vehicle by applying a convolutional neural network (CNN) to the range-angle detection result was proposed in [10]. In our work, we use a You Only Look Once (YOLO)-based network [11], one of the CNN-based classifiers for target detection and classification.

Research on applying the YOLO network to radar sensor data has been actively conducted in recent years [12,13,14,15,16,17]. In [12,13], the basic YOLO network-based object detection was performed in the range-velocity domain. In addition, the target classification in the two-dimensional (2D) range domain using YOLO networks were proposed in [14,15]. In recent studies, more advanced versions of YOLO networks were applied to radar sensor data to perform effective object detection [16,17]. Generally, the YOLO network is used to locate an object and classify its type in a given red, green, and blue (RGB) image. To combine the YOLO network with a high-resolution radar system, a method of converting an object detected as a point cloud into a 2D image form is required. Thus, we propose a method of storing the radar detection result as an image through lossy compression, which enables the network to extract the features of the object effectively. Through the proposed technique, we can generate input data in a form suitable for training the YOLO-based network.

Finally, we evaluate the performance of the proposed method using radar sensor data acquired in the test field. In our experiment, we use a frequency-modulated continuous wave (FMCW) radar system with the center frequency of 62 GHz and the bandwidth of 3 GHz. This frequency band is being considered important for joint radar and communications in recent years [18,19]. The radar sensor data are obtained for cases where a person, a cyclist, and a car move in various directions, and then the type and moving direction of each object are determined using the YOLO network-based classifier. Moreover, the performance of the pre-trained classifier is verified with new data that have not been used for the network training. The proposed method can play an important role in a bad environment where the object recognition performance of the camera sensor is severely degraded.

In summary, the main contributions of this paper are:

- We obtained the point cloud-based object detection result using a high-resolution radar system, and proposed a method to convert it into an image format suitable for training the CNN-based classifier.
- Based on the high-resolution radar sensor data, we designed a deep learning-based classifier that can determine the type of detected object and estimate its moving direction as well. In conventional studies, classifiers that perform only a single purpose have been proposed.
- Our proposed method is different from the target classification methods using the range and velocity information of an object [12,13], because the proposed target classification method is performed based on the overall shape of the object.
- In conventional radar systems, target detection, point clustering, and target tracking are sequentially performed to estimate the moving direction of an object. The proposed method can determine the moving direction of the object by using only the target detection results.

The remainder of this paper is organized as follows. First, the basic principles of the millimeter-wave FMCW radar sensor are introduced in Section 2. Then, Section 3 describes the conversion of radar detection results into images. In Section 4, the proposed simultaneous target classification and moving direction estimation method is explained and its performance is evaluated. Finally, we conclude this paper in Section 5.

## 2. Basic Principles of Millimeter-Wave FMCW Radar System

### 2.1. Millimeter-Wave Band FMCW Radar Sensor

**Figure 1:**
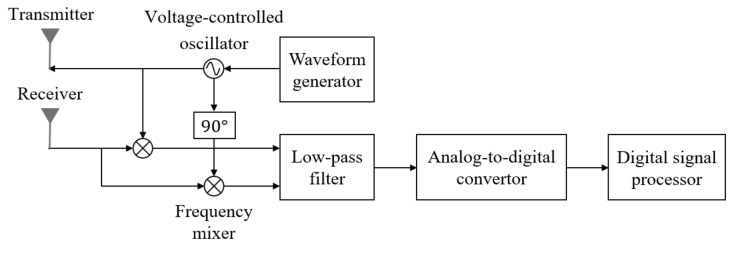
Configuration of the millimeter-wave band FMCW radar system.

**Figure 2:**
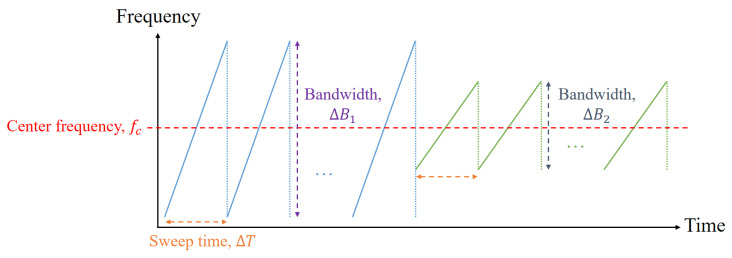
Waveforms transmitted from the FMCW radar system.

In this study, we used an FMCW radar system operating in the millimeter wave band, and its configuration is shown in Figure 1. In this radar system, a series of waveforms whose frequency increases linearly with time are transmitted, as shown in Figure 2. In the figure, $f_{c}$ represents the center frequency of the transmitted signal, and ΔT and $\Delta B_{m}\;\left(m=1,\;2\right)$ are the sweep time and bandwidth of each waveform, as shown in Figure 2. All waveforms use the same center frequency, but the first $N_{1}$ waveforms and the next $N_{2}$ waveforms use different bandwidths. Thus, the *n*-th transmitted waveform in (n−1)ΔT<t<nΔT can be expressed as

(1) $$\begin{array}{c} x_{n}\left(t\right)=A_{x}\exp\left(j\left(2\pi \left(f_{c}-\frac{2n-1}{2}\Delta B_{n}\right)t+2\pi \frac{\Delta B_{n}}{\Delta T}t^{2}+\phi_{n}\right)\right), \end{array}$$

where $A_{x}$ and $\phi_{n}$ represent the amplitude and phase offset of the *n*-th transmitted waveform.

The specifications of the radar sensor we used are summarized in Table 1. In general, the range resolution of the FMCW radar system is determined by the bandwidth, which can be calculated as $\Delta r=\frac{c}{2B_{n}}$ [20]. As given in the table, a single target can be detected as multiple points because the range resolution is in units of several centimeters. In addition, the signal processing cycle of our radar system is 50 ms, which means that one detection result is generated every 50 ms.

### 2.2. Distance, Velocity, and Angle Estimation in FMCW Radar System

The transmitted signal in (1) is reflected by targets in the radar’s field of view. To extract both amplitude and phase information from the received signal, it is passed through the in-phase and quadrature (IQ) modulator. The IQ-modulated received signal can be expressed as

(2) $$\begin{array}{c} y_{n}\left(t\right)=\sum_{k=1}^{K}A_{y,\;k}\exp\left(j\left(2\pi \left(f_{c}-\frac{2n-1}{2}\Delta B_{n}\right)\left(t-t_{d,\;k}\right)+2\pi \frac{\Delta B_{n}}{\Delta T}{\left(t-t_{d,\;k}\right)}^{2}+\phi_{n}\right)\right), \end{array}$$

where $A_{y,\;k}$ denotes the amplitude of the received signal reflected from the *k*-th (k=1,…,K) target. In addition, $t_{d,\;k}$ denotes the time delay caused by the distance between the radar and the *k*-th target.

Then, $y_{n}\left(t\right)$ is multiplied by $x_{n}\left(t\right)$, and the output passes through a low-pass filter (LPF), as shown in Figure 1. Finally, a down-converted baseband signal is obtained, which is expressed as

(3) $$\begin{array}{c} m_{n}\left(t\right)={\left\{x_{n}\left(t\right)y_{n}\left(t\right)\right\}}_{\mathrm{LPF}}, \end{array}$$

where ${\left\{\cdot \right\}}_{\mathrm{LPF}}$ represents the output of the LPF. This baseband signal consists of the sum of sinusoids, and each sinusoid contains distance and velocity information for each object. By applying a Fourier transform to a set of $m_{n}\left(t\right)$, the distance to the object and the velocity of the object can be estimated [21]. For example, in the short-range detection mode, distance and velocity information of several objects can be estimated by applying a 2D Fourier transform to $m_{s}=\left[m_{1}\left(t\right),\;m_{2}\left(t\right),\;\ldots ,\;m_{N1}\left(t\right)\right]$.

Moreover, to find the exact position of the object, the angle between the radar sensor and the object must be identified. In the automotive radar system, multiple receiving antenna elements are widely used to estimate the angle information of an object [22]. To improve the angular resolution, our radar system adopts a multiple-input and multiple-output (MIMO) antenna system consists of several transmit and receiving antenna elements [6], as given in Table 1. These antenna elements are arranged in the horizontal direction for azimuth angle estimation. The angle of the object can be estimated from the phase difference of the signals incident on the horizontally arranged antenna element [23]. In this study, we use a delay-and-sum beamforming method [24] to estimate the angle information of multiple targets [25].

## 3. Target Image Generation in Radar System

In this section, the radar signal measurement environments and experimental scenarios are described. In addition, a method of converting the object detection result into an image form suitable for training the YOLO network is proposed.

### 3.1. Measurement Environment

**Figure 3:**
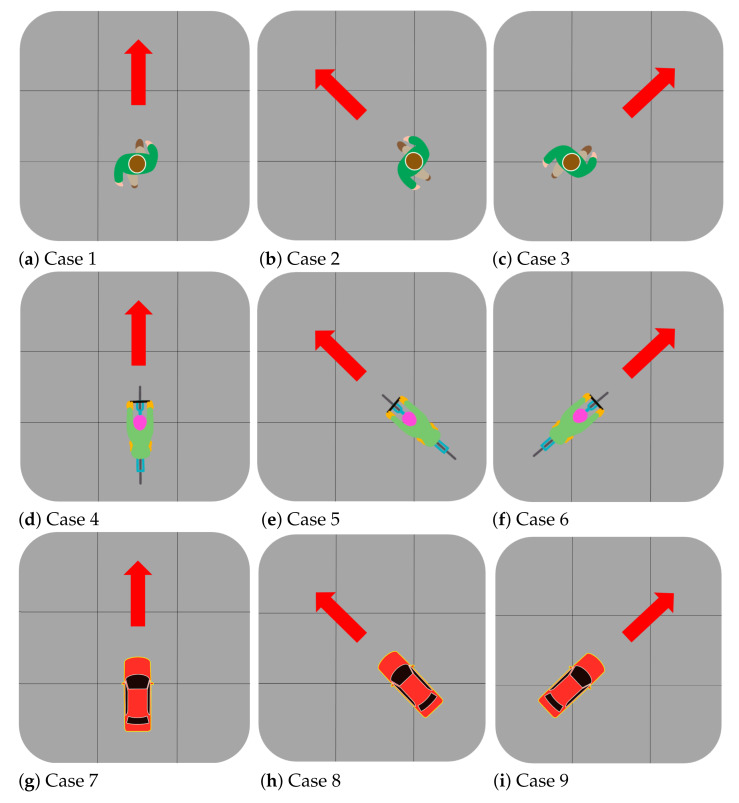
Experimental scenarios for a pedestrian, a cyclist, and a car: Cases 1 to 9.

**Figure 4:**
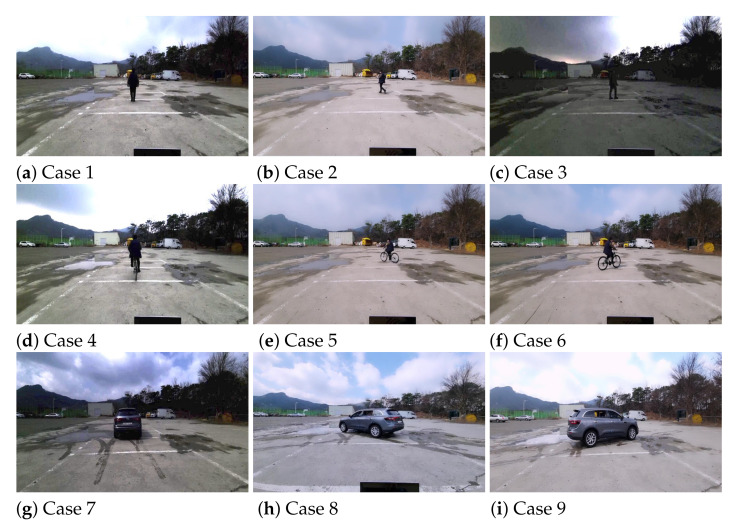
Radar signal measurements in the test field: Cases 1 to 9.

With the FMCW radar sensor described in Section 2, radar signal measurements were conducted. In this experiment, we divided the measurement results into 9 representative cases (i.e., Cases 1 to 9), as shown in Figure 3. Each object went straight, moved from left to right, and moved from right to left, and the same experiments were repeated by changing the distance between the object and the radar. A pedestrian, a cyclist, and a car moved in the test field as shown in Figure 4, and reflected radar signals were acquired and stored for each case.

### 3.2. Radar Detection Result in 2D Distance Plane

As explained in Section 2.2, we can estimate the distance, velocity, and angle information of the target from the received radar signal. The information of the *k*-th target can be expressed in the 2D *x*-*y* distance plane as follows:

(4) $$\begin{array}{c} {\left[x_{k},\;y_{k}\right]}^{T}={\left[{\hat{d}}_{k}\sin{\hat{\theta }}_{k},\;{\hat{d}}_{k}\cos{\hat{\theta }}_{k}\right]}^{T}, \end{array}$$

where ${\hat{d}}_{k}$ and ${\hat{\theta }}_{k}$ are the estimated distance and angle between the radar and the *k*-th target, respectively. However, in a high-resolution radar system, a single target is detected as multiple points. That is, the *k*-th target is detected as points ${\left[x_{k_{p}},\;y_{k_{p}}\right]}^{T}\;\left(p=1,\;2,\;\ldots ,\;P_{k}\right)$, where $P_{k}$ is the number of points derived from the *k*-th target.

In previous studies [12,13,15], the type of object was determined with a single detection result. However, it is difficult to extract information about the movement of the target from the single detection result. Thus, to understand the change in the moving direction of the target over time, we use the accumulated detection results. If the accumulated detection results are used, the velocity information of the target is also reflected and the probability of false object detection can be reduced. As given in Table 1, target detection results are generated every 50 ms in our radar system. Therefore, if $N_{D}$ detection results are accumulated, information on the movement change of the target for $N_{D}\times 50$ ms can be identified. In this study, we accumulated 15 consecutive detection results, which means that $N_{D}$ becomes 15 and $N_{D}\times 50$ becomes 0.75 s.

**Figure 5:**
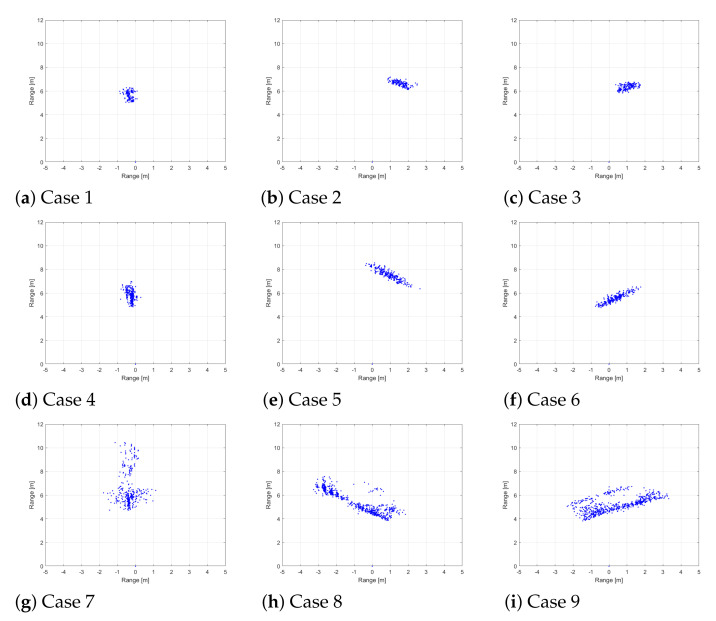
Accumulated detection results in the 2D distance plane: (**a**) when a pedestrian goes straight (**b**) when a pedestrian moves from right to left (**c**) when a pedestrian moves from left to right (**d**) when a cyclist goes straight (**e**) when a cyclist moves from right to left (**f**) when a cyclist moves from left to right (**g**) when a car goes straight (**h**) when a car moves from right to left (**i**) when a car moves from left to right.

Figure 5 shows the accumulated radar detection results for 0.75 s for a pedestrian, a cyclist, and a car, respectively. As shown in the figure, each target is detected as a number of points because the range resolution of our radar sensor is in units of several centimeters. From a pedestrian to a car, each target is detected as more points because the physical size of the target increases. In addition, the movement information of the target is reflected in the accumulated detection result.

### 3.3. Target Image Generation

In general, RGB images are widely used as input data for CNN-based image classifiers. However, because the detection results in Figure 5 are expressed as monochromatic point clouds, they are not suitable as input data for the CNN-based classifier. Therefore, it is essential to convert the radar detection result into an appropriate input format.

As a solution, we save the detection result in the joint photographic experts group (JPEG) format, one of the lossy compression method [26]. By storing the detection result through the lossy compression method, we can force color difference to the monochromatic detection result. For example, where there are many blue points, the distortion of the RGB values is small, but in the part where there are few blue points, the RGB values are severely distorted. Thus, if the area where the points are concentrated is defined as the central area, the points in the central area are expressed close to black and blue, and the points in the surrounding area are expressed in red, yellow, green, pink, and cyan. Therefore, through the proposed transformation method, information on the edge of the object and the number of points constituting the object is stored as RGB information.

**Figure 6:**
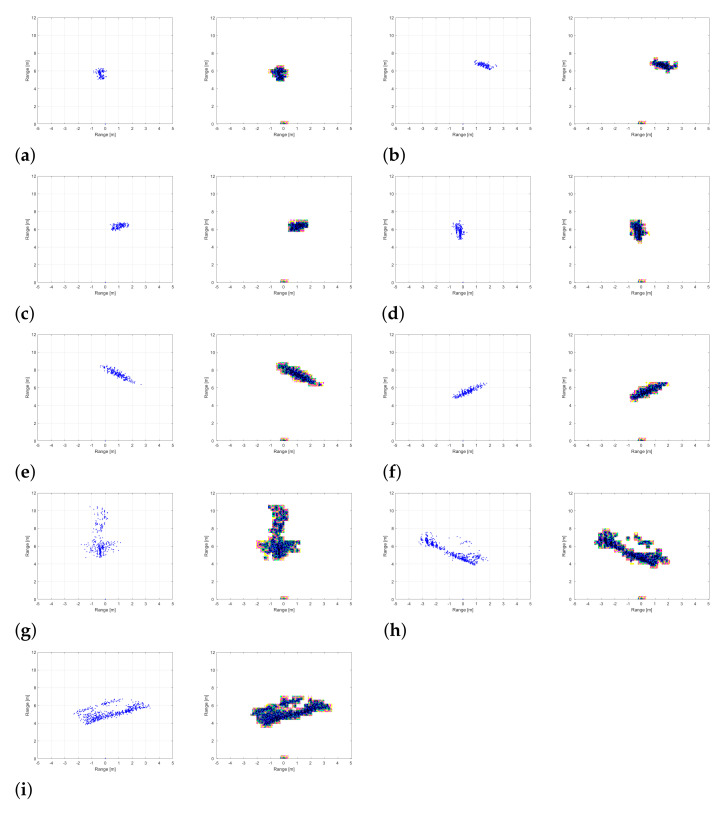
Regenerated detection results: (**a**) when a pedestrian goes straight (**b**) when a pedestrian moves from right to left (**c**) when a pedestrian moves from left to right (**d**) when a cyclist goes straight (**e**) when a cyclist moves from right to left (**f**) when a cyclist moves from left to right (**g**) when a car goes straight (**h**) when a car moves from right to left (**i**) when a car moves from left to right.

Figure 6 shows the object detection results converted into images suitable for training. As shown in the figure, near the center of each target is represented by dark colors, and the edges of each target are represented by relatively light colors. Therefore, by converting the detection result into an image format with the lossy compression, information on the width and length of the object is effectively reflected in RGB values.

## 4. Proposed Simultaneous Target Classification and Moving Direction Estimation

In this section, a method of classifying the type of target and determining its moving direction with the regenerated object detection results is introduced. Then, the performance of the proposed method is evaluated based on the radar sensor data acquired from the test field. In addition, new data that have not been used for network training are also used to validate the feasibility of our proposed method.

### 4.1. Structure of YOLO Network for Radar Target Identification

**Figure 7:**
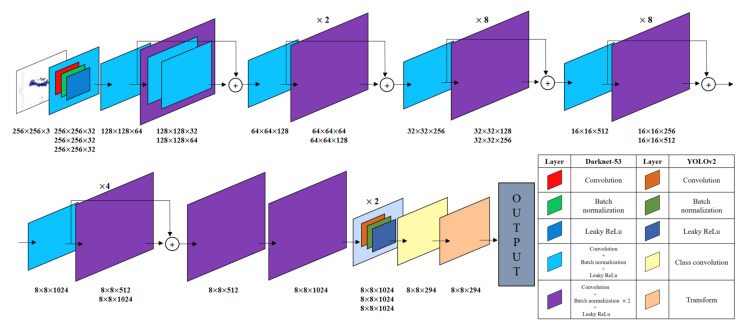
Proposed network for the target classification and moving direction estimation.

In this work, a YOLO-based network is used to identify the type and moving direction of the target, which is described in Figure 7. As shown in the figure, the Darknet-53 [27] was used in the feature extraction stage, and the YOLOv2 [28] was used in the classification stage. In the feature extraction network, the converted input image passes through multiple blocks, each of which consists of layers for convolution, batch normalization, and activation. As an activation function, a leaky rectified linear unit (ReLu) is used. The sizes of the filters used in each layer are also indicated in Figure 7. Through this process, target features are extracted at multiple scales. Then, the YOLO network performs target identification based on the features extracted from the Darknet-53. Finally, the proposed network finds the location and size of the target in each input image, and determines it as one of nine cases (e.g., Cases 1 to 9).

**Figure 8:**
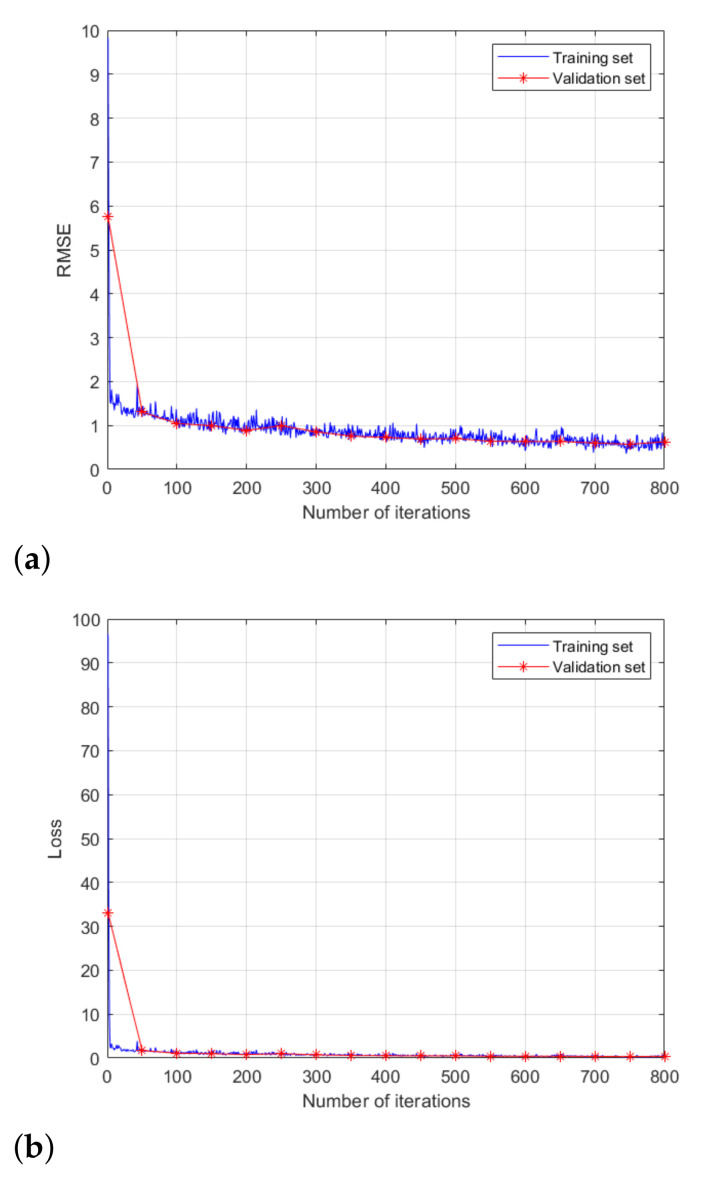
Training and validation of the network: (**a**) RMSE (**b**) loss value.

Table 2 summarizes the number of input image data used for training, validation, and test. For training, validation, and test data sets, 70%, 10%, and 20% of total input images were used, respectively. In addition, the parameter values used when training the network are summarized in Table 3. With the given parameter values, we trained the network in Figure 7, and Figure 8 shows the root-mean-square error (RMSE) and and loss values for the data set used for network training and validation. After 500 iterations, the network performance is stabilized, and the RMSE and loss values converge to specific values.

### 4.2. Performance Evaluation

In our network, when the accumulated radar detection result converted into an RGB image is input to the trained network, the location, type and direction of movement of the target are determined immediately, which means that simultaneous target detection and classification is possible. In other words, when the detected points are scattered in the accumulated detection result, the YOLO-based network immediately finds points that correspond to meaningful targets.

**Figure 9:**
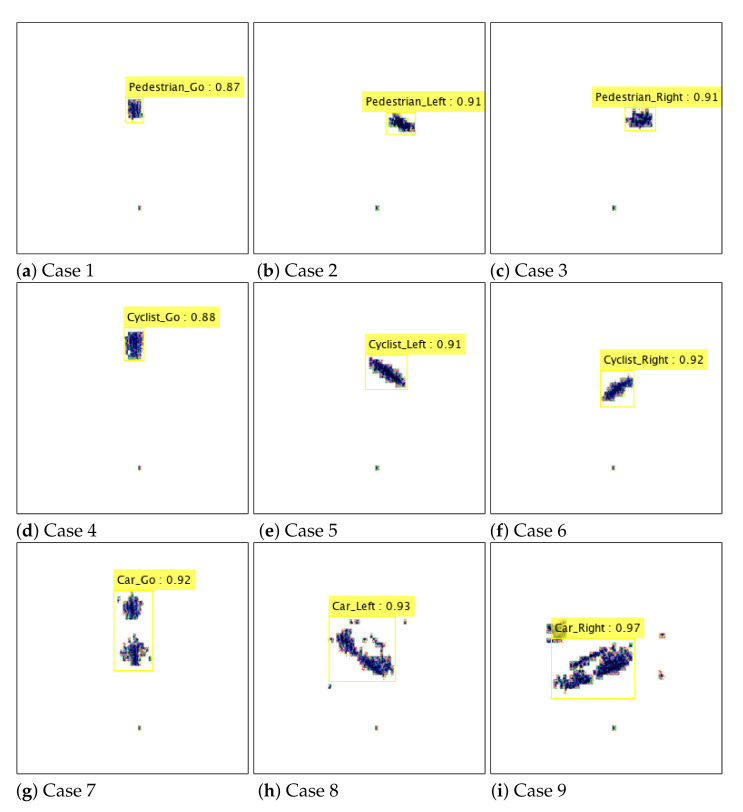
Instantaneous target identification results: Cases 1 to 9.

Figure 9 shows the instantaneous target identification results through our proposed network. After network training is finished, the processing speed for new input is fast because only simple addition and multiplication operations are performed inside the network. The yellow box in the figure shows the expected type and moving direction of the detected object. In addition, the number in the box indicates the probability of how close the detected object is to the expected case. As shown in the figure, the type of detected target and its direction of movement are simultaneously determined in each image with high accuracy.

In addition, Table 4 is a confusion matrix showing classification results for a total of 9 cases. In our data set, most of the classification errors occurred when identifying the movement of a pedestrian and a cyclist. In other words, the trained network confuses Cases 2 and 5, and Cases 3 and 6 the most. As shown in Figure 6, the widths of a pedestrian and a cyclist detected by the radar are similar, and the main difference between the two objects exists in the length of the point cloud. As input to the network, we used the accumulated radar detection results. Therefore, a fast-moving pedestrian and a slow-moving cyclist can show similar patterns in detection results, which can degrade the classification performance of the network. On the other hand, in the case of a car, its type and movement is identified with very high accuracy, because the number of detected points and the size of the point cloud are larger than those of the pedestrian and cyclist.

We also compared the performance of the proposed method with that of the moving direction estimation method using a simple CNN [10]. When the same data were used as input, the average estimation accuracy was 91.4%. Unlike the YOLO network, all input images need to be set to the same size to train a CNN. In addition, the location of an object cannot be automatically found when the simple CNN-based method is used, which was possible with the YOLO-based network. Thus, the YOLO network-based moving direction estimation is more efficient in terms of target detection and classification.

### 4.3. Performance Evaluation in New Environments

**Figure 10:**
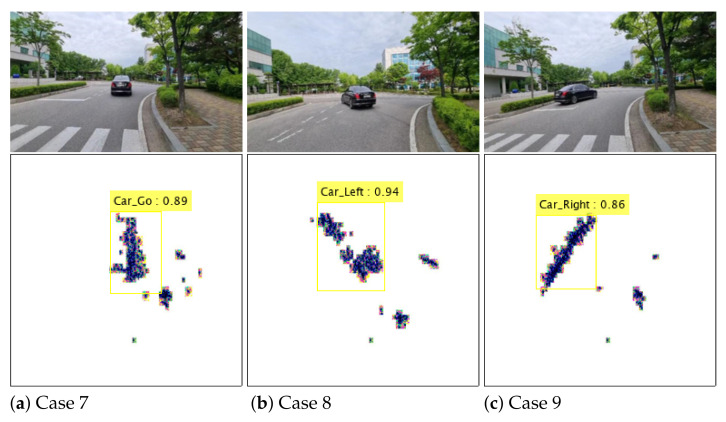
Instantaneous target identification results in new environments: Cases 7 to 9.

We also checked how the classification performance of the trained YOLO network changes for new data sets other than the existing data sets. Thus, we acquired data in a new environment and put the data into the pre-trained network to evaluate its performance. Figure 10 shows the result of immediately discriminating the type and moving direction of the detected object by the pre-trained network. As given in the yellow box, although they are new data that have not been used for training, the pre-trained network shows quite high identification accuracy.

In the newly acquired data set, the type and moving direction of the car were determined with an average of 85% accuracy. We expected that higher recognition accuracy can be obtained by increasing the amount of radar sensor data used for network training. In addition, as shown in Figure 10, the tree on the right side of the vehicle is continuously detected, but the YOLO-based network determined it as a meaningless target. By accumulating experimental data for various types of targets on the road and labeling them, a network having more stable classification performance can be trained.

## 5. Conclusions

In this paper, we proposed a method of classifying the type of target and estimating its moving direction in the millimeter-wave FMCW radar system. In our method, lossy compression was used to convert the object detection results of the radar sensor into an RGB image format. Then, we designed and trained the YOLO-based classifier with the input images to determine the type and moving direction of a pedestrian, a cyclist, and a car. The trained YOLO network determined the type and direction of the target with an accuracy of over 95%. In addition, for the new data set that were not used for training, the proposed method showed more than 85% recognition and classification accuracy. The results of this study can be used to find out the heading direction of the vehicle through the overall shape of the detected points, which can help predict the next movement of the detected vehicle.

## Figures and Tables

**Table 1:** Specifications of our FMCW Radar Sensor.

Parameters	Detection Mode	
	Short-Range Mode	Long-Range Mode
Center frequency, $f_{c}$ (GHz)	62	
Bandwidth, ΔB (GHz)	3	1.5
Range resolution, Δr (cm)	5	10
Transmit and receiving antenna elements	1 × 4	2 × 4
Sweep time, ΔT (μs)	150	
The number of waveforms, $N_{1}$ and $N_{2}$	128	
Signal processing cycle (ms)	50	

**Table 2:** The Number of Input Image Data Used for Training, Validation, and Test.

Case	Training (%)	Validation (%)	Test (%)	Total Number of Images
Case 1	70.8	10	19.2	250
Case 2	74.5	7.7	17.8	220
Case 3	71.6	9.8	18.6	225
Case 4	71	13.4	15.6	500
Case 5	71.4	9	19.6	500
Case 6	67.4	9.6	23	500
Case 7	71.2	10	18.8	309
Case 8	68.2	7.8	24	462
Case 9	67.1	11.6	21.3	371

**Table 3:** Parameter Values Used in YOLO-based Network.

Parameter	Value
Batch size	128
Width	256 (pixels)
Height	256 (pixels)
Channels	3 (R, G, B)
Learning rate	0.001
Momentum	0.9
Max epochs	30
Iterations	4350

**Table 4:** Confusion Matrix for Cases 1 to 9.

Actual Case	Predicted Case (%)								
	Case 1	Case 2	Case 3	Case 4	Case 5	Case 6	Case 7	Case 8	Case 9
Case 1	98.0	0	0	2	0	0	0	0	0
Case 2	0	100.0	0	0	0	0	0	0	0
Case 3	0	0	95.3	0	0	4.7	0	0	0
Case 4	0	0	0	100.0	0	0	0	0	0
Case 5	0	6.12	0	0	93.88	0	0	0	0
Case 6	0	0	1.74	0	0	98.26	0	0	0
Case 7	0	0	0	0	0	0	100.0	0	0
Case 8	0	0	0	0	0	0	0	100.0	0
Case 9	0	0	0	0	0	0	0	0	100.0

## Data Availability

Data sharing not applicable.

## Abbreviations

The following abbreviations are used in this manuscript:

2D	Two-dimensional
CNN	Convolutional neural network
FMCW	Frequency-modulated continuous wave
IQ	In-phase and quadrature
JPEG	Joint photographic experts group
LPF	Low-pass filter
MIMO	Multiple-input and multiple-output
RGB	Red, green, and blue
RMSE	Root-mean-square error
YOLO	You only look once
